# State-Specific Synthetic Estimates of Health Status Groups Among Inactive Older Adults With Self-Reported Diabetes, 2000-2009

**Published:** 2012-04-19

**Authors:** Karen A. Kirtland, Matthew M. Zack, Carl J. Caspersen

**Affiliations:** Northrop Grumman; Centers for Disease Control and Prevention, Atlanta, Georgia; Centers for Disease Control and Prevention, Atlanta, Georgia

## Abstract

**Introduction:**

Physical activity helps diabetic older adults who have physical impairments or comorbid conditions to control their disease. To enable state planners to select physical activity programs for these adults, we calculated synthetic state-specific estimates of inactive older adults with diabetes, categorized by defined health status groups.

**Methods:**

Using data from the 2000 through 2009 National Health Interview Survey (NHIS) and the Behavioral Risk Factor Surveillance System (BRFSS), we calculated synthetic state-specific estimates of inactive adults with diabetes who were aged 50 years or older for 5 mutually exclusive health status groups: 1) homebound, 2) frail (functional difficulty in walking one-fourth mile, climbing 10 steps, standing for 2 hours, and stooping, bending, and kneeling), 3) functionally impaired (difficulty in 1 to 3 of these functions), 4) having 1 or more comorbid conditions (with no functional impairments), and 5) healthy (no impairments or comorbid conditions). We combined NHIS regional proportions for the health status groups of inactive, older diabetic adults with BRFSS data of older diabetic adults to estimate state-specific proportions and totals.

**Results:**

State-specific estimates of health status groups among all older adults ranged from 2.2% to 3.0% for homebound, 5.8% to 8.8% for frail, 20.1% to 26.1% for impaired, 34.9% to 43.7% for having comorbid conditions, and 4.0% to 6.9% for healthy; the remainder were older active diabetic adults. Except for the homebound, the percentages in these health status groups varied significantly by region and state.

**Conclusion:**

These state-specific estimates correspond to existing physical activity programs to match certain health status characteristics of groups and may be useful to program planners to meet the needs of inactive, older diabetic adults.

## Introduction

Type 2 diabetes is a major cause of illness and death in the United States, especially among older adults, resulting in human and economic costs (1). In 2010, a total of 10.9 million Americans aged 65 years or older had either diagnosed or undiagnosed diabetes (1). Among these older adults, approximately 390,000 new diabetes cases were diagnosed in 2010 (1), and diagnosed diabetes cases will likely increase to 26.7 million in 2050 from 6.3 million in 2005 (2).

Adults with diabetes are at 1.8 to 2.4 times greater risk of various forms of disability than their nondiabetic counterparts (3). Older adults with diabetes are more likely than those without the disease to have less muscle strength, poorer muscle quality, and accelerated muscle loss (4). Older diabetic adults may respond favorably to exercise programs that improve glycemic control (5,6). Among older diabetic adults, resistance training can improve muscle strength and glycemic control (7,8). In addition, resistance training can prevent or delay the development of sarcopenia and osteoporosis and improve functional capacity in tasks of daily living among older adults with diabetes (6).

Older diabetic adults are more likely to have a sedentary lifestyle (9) and are less likely to comply with physical activity recommendations (10). A recently developed *Reference Guide of Physical Activity for Older Adults* (PARG) identifies and promotes physical activity programs for older adults (11) whose health status ranges from homebound to healthy.

Our objective was to provide state-specific estimates of inactive, older diabetic adults, categorized by defined health status groups. Knowing state-specific proportions in these groups may help planners select physical activity programs for their states (11).

## Methods

Using cross-sectional data from the 2000 through 2009 National Health Interview Survey (NHIS), we identified health status categories to correspond to the health status groups defined by the PARG (11) (Box). Health status data for these categories are unavailable in the 2000 through 2009 Behavioral Risk Factor Surveillance System (BRFSS); therefore, we applied the NHIS regional estimates of the health status categories among inactive, older, diabetic adults to state-specific BRFSS estimates of older diabetic adults to create state estimates for each health status group.

### Data sources

NHIS and BRFSS conduct annual nationally representative surveys of the health of the civilian, noninstitutionalized US population. NHIS incorporates a household interview in which trained staff annually visit approximately 35,000 households containing approximately 87,500 people (www.cdc.gov/nchs/nhis/about nhis.htm). The response rates for its sample adult questionnaire were 72.1% in 2000 and 65.4% in 2009.

BRFSS is a state-based telephone survey of more than 350,000 adults (www.cdc.gov/brfss/index.htm#content_area). In 2000, overall response rates ranged from 28.8% to 71.8% (median, 48.9%) and cooperation rates from 35.5% to 77.7% (median, 53.2%) (12). In 2009, response rates ranged from 19.2% to 61.8% (median, 34.9%) and cooperation rates from 55.5% to 88.0% (median, 75.0%) (13).

### Study sample and variables

**Table:** 

**Box. Health status groups and the 2000-2009 NHIS items**	
**Health Status Group**	**Specific Value or Combination From NHIS Survey Item**
Homebound^a^	Reported at least 180 bed days in the past year
Frail^a^	Difficulty in all 4 functions: Walking one-fourth mile Climbing 10 steps Standing for 2 hours Stooping, bending, or kneeling
Impaired^a^	Difficulty in 1 to 3 functions: Walking one-fourth mile Climbing 10 steps Standing for 2 hours Stooping, bending, or kneeling
Comorbid condition(s)	Having 1 or more of the following: Arthritis Cancer CVD (CHD, angina, heart attack/condition/disease) Stroke Hypertension Respiratory illness (emphysema, asthma) Problems seeing with corrective lenses
Healthy	Not being homebound Not being frail None of 4 impairments None of 7 comorbid conditions

Abbreviations: NHIS, National Health Interview Survey; CVD, cardiovascular disease; CHD, coronary heart disease.

a The presence of 1 or more comorbid conditions used to devise the fourth health status category may also occur for the first 3 health status groups. However, neither of the last 2 groups have any of the 4 functional difficulties.

From the NHIS data, we identified inactive older diabetic adults as those aged 50 or older who had been told they had diabetes and indicated they had never engaged or were unable to engage in leisure-time moderate or vigorous activities and categorized them by US census region (Northwest, Midwest, South, and West). We grouped respondents by their difficulty in performing the following 6 functions: walking one-fourth mile; climbing up 10 steps; standing for 2 hours; stooping, bending, or kneeling; reaching above their head; and lifting or carrying 10 pounds. Because few respondents had difficulty with reaching, lifting, or carrying, we defined 2 of our health status groups on the basis of the respondents' reported difficulty with only the remaining 4 functions. Using the most restrictive health status group (homebound) as a starting point, we assigned each respondent to only 1 of the 5 health status groups (Box).

We calculated synthetic state-specific estimates of older, physically inactive diabetic adults in each health status group by multiplying the region-specific proportion of each health status group (2000-2009 NHIS) by the state-specific proportion of older diabetic adults (2000-2009 BRFSS).

To estimate the standard error of these state-specific synthetic proportions, we applied a simple exact formula for the variance of each proportion (14). The final state-specific synthetic proportions do not total 100% because the estimates represent only the BRFSS values for inactive diabetic adults. The remainder would represent active diabetic adults from BRFSS.

### Statistical analysis

We conducted all analyses using SAS-callable SUDAAN version 9.1 (RTI International, Research Triangle Park, North Carolina) and accounted for the complex survey design, nonresponse, and weighting to represent the US population. National proportions and 95% confidence intervals (CIs) for the 5 health status groups were stratified by the 4 census regions. We calculated nonoverlapping 95% confidence intervals to determine whether proportion estimates among regions and states differed (15).

## Results

BRFSS percentage distributions by age and sex resembled those of NHIS for similar years but differed by education and race (Table). In the 2000 through 2009 NHIS, the overall percentage of inactive older diabetic adults who were homebound was approximately 3% (95% CI, 3.4% (95% CI, 2.9%-3.9%); frail, 9.6% (95% CI, 8.9%-10.3%); impaired, 29.8% (95% CI, 28.7%-30.9%); having a comorbid condition, 50.5% (95% CI, 49.4%-51.8%); and healthy, 6.7% (95% CI, 6.1%-7.3%).

**Figure 1.**
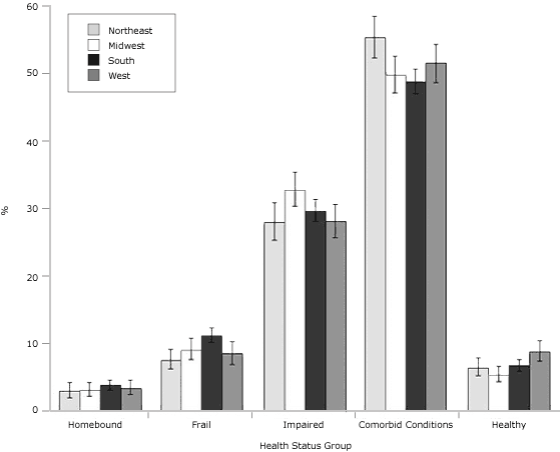
Regional estimates of health status groups among physically inactive diabetic adults aged 50 years or older, National Health Interview Survey, 2000-2009. Error bars represent 95% confidence intervals. Conditions defined as homebound: ≥180 bed days in last year; frail: functional difficulty in walking one-fourth mile, climbing 10 steps, standing for 2 hours, and stooping, bending, and kneeling; impaired: difficulty in 1 to 3 of these functions; comorbid conditions: ≥1, with no functional impairments; and healthy: no impairments or comorbid conditions.

The NHIS regional percentages in the health status groups varied significantly for the frail, people with comorbid conditions, and the healthy (Figure 1). Specifically, the percentages for the frail status ranged from 8% in the Northeast to 11% in the South. Forty-nine percent in the South had at least 1 comorbid condition, compared with 55% in the Northeast. The percentage of the healthy ranged from 5% in the Midwest to 9% in the West.

**Table:** 

Region	% (95% CI)				

	Homebound	Frail	Impaired	≥1 Comorbid Condition	Healthy
**Northeast**	2.9 (1.9-4.2)	7.5 (6.2-9.1)	27.9 (25.2-30.8)	55.3 (52.3-58.4)	6.4 (5.2-7.8)
**Midwest**	3.1 (2.2-4.2)	9.0 (7.6-10.8)	32.7 (30.3-35.3)	49.8 (47.1-52.5)	5.3 (4.3-6.6)
**South**	3.8 (3.1-4.6)	11.1 (10.1-12.3)	29.6 (28.0-31.3)	48.8 (47.0-50.6)	6.7 (5.8-7.6)
**West**	3.3 (2.4-4.6)	8.5 (6.9-10.3)	28.0 (25.6-30.6)	51.5 (48.6-54.3)	8.7 (7.3-10.4)

None of the state-specific percentages of the homebound differed significantly among the states (range, 2.2%-3.0%) (Figure 2a). State-specific percentages by health status group varied significantly for the frail (6% in Massachusetts to 9% in Florida) (Figure 2b); the impaired (20% in Alaska to 26% in Iowa) (Figure 2c); people with at least 1 comorbid condition (35% in Texas to 44% in Pennsylvania) (Figure 2d); and people who are otherwise healthy (4% in Michigan and Ohio to 7% in Montana) (Figure 2e).

The estimated total of adults in each health status group tended to be lowest in the least populous state, Alaska, and highest in the most populous state, California (Appendices A and B). For Alaska, estimates ranged from 396 homebound people to 6,157 people having comorbid conditions (Figures 2a-2e). In California, estimates ranged from 35,598 homebound people to 552,807 people having comorbid conditions.

**Figure 2a.**
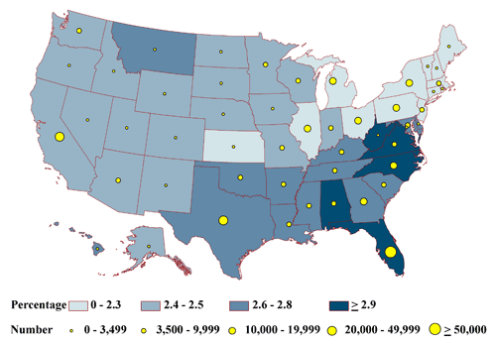
State-specific proportions and numbers of physically inactive diabetic adults aged 50 years or older who are homebound, 2000-2009 NHIS and 2000-2009 BRFSS. Proportions are classified by quartiles and numbers by the 5 groupings indicated. Homebound is defined as reporting at least 180 bed days in the past year.

**Figure 2b.**
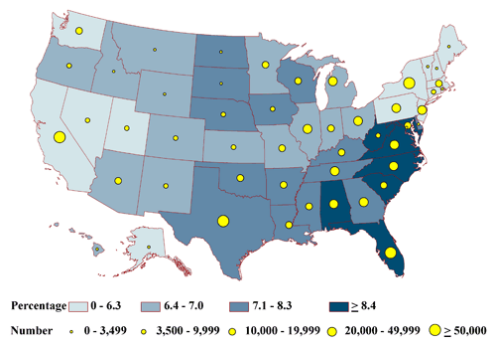
State-specific proportions and numbers of physically inactive diabetic adults aged 50 years or older who are frail, 2000-2009 NHIS and 2000-2009 BRFSS. Proportions are classified by quartiles and numbers by the 5 groupings indicated. Frail is defined as reporting difficulty with all 4 of the following functions: walking one-fourth mile, climbing 10 steps, standing for 2 hours, and stooping, bending, or kneeling.

**Figure 2c.**
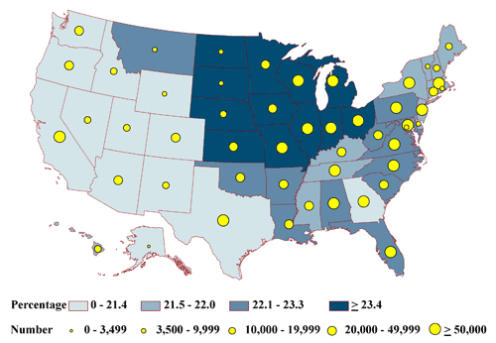
State-specific proportions and numbers of physically inactive diabetic adults aged 50 years or older who are impaired, 2000-2009 NHIS and 2000-2009 BRFSS. Proportions are classified by quartiles and numbers by the 5 groupings indicated. Impaired is defined as reporting difficulty with 1 to 3 of the following functions: walking one-fourth mile, climbing 10 steps, standing for 2 hours, or stooping, bending, or kneeling.

**Figure 2d.**
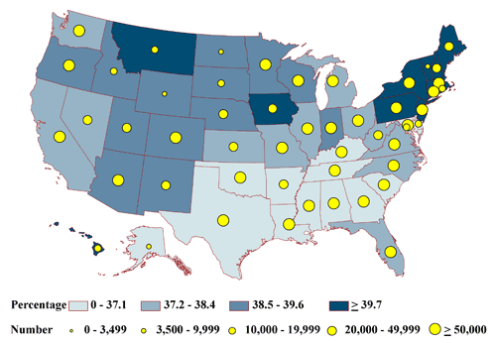
State-specific proportions and numbers of physically inactive diabetic adults aged 50 years or older who have 1 or more comorbid conditions, 2000-2009 NHIS and 2000-2009 BRFSS. Proportions are classified by quartiles and numbers by the 5 groupings indicated. Comorbid condition is defined as not meeting criteria for homebound, frail, or impaired and reporting at least 1 of the following chronic conditions: arthritis, hypertension, cancer, stroke, respiratory illness (emphysema, asthma), cardiovascular disease (coronary heart disease, angina pectoris, heart attack, heart condition/disease), or trouble seeing even with corrective lenses.

**Figure 2e.**
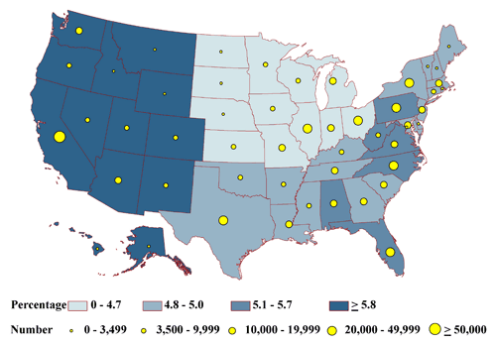
State-specific proportions and numbers of physically inactive diabetic adults aged 50 years or older who are otherwise healthy, 2000-2009 NHIS and 2000-2009 BRFSS. Proportions are classified by quartiles and numbers by the 5 groupings indicated. Healthy is defined as not meeting the criteria for homebound, frail, impaired, or having comorbid conditions.

## Discussion

This study provides the first set of state-specific estimates of the percentages and the number of inactive, older, diabetic adults in 5 health status groups who may benefit from physical activity programs. These estimates may facilitate planning of physical activity programs for diabetic older adults with varying functional health status (11).

The highest percentage of the homebound and the frail corresponds with higher diagnosed diabetes rates in Southern states (16), and the highest percentage of the healthy corresponds with lower diagnosed diabetes rates in Western states (16). Our estimates may prove valuable to state planners because the functional burden among older adults with diabetes is greater than that for those without diabetes (17). People with diabetes in the United States are 2 to 3 times more likely than older, nondiabetic adults to have various forms of disability, including reduced lower-extremity mobility and reduced general physical activities (3). Followed prospectively, older diabetic adults have a 42% higher risk for developing disability than older nondiabetic adults (18). Diabetes often increases the risk for many other comorbid conditions (19) that may lead to future declines in physical function (18,20-22) and poorer health-related quality of life (23).

Physical activity programs can help older diabetic adults by improving glycemic control (24,25), which reduces the development of microvascular and other complications (5,25) and other comorbid conditions such as high blood pressure and dyslipidemia (5,25). Appropriate physical activity programs can restore function among homebound, frail, and impaired adults; control comorbid conditions; and help adults who have diabetes but are otherwise healthy to resist progression to a more severe health status. Recent guidelines for older adults call for individualized patient care and education, the prevention and control of cardiovascular risk factors, and screening for combinations of comorbid conditions, while also regulating glycemic status to prevent and control microvascular complications (26). These guidelines may require balancing glycemic targets, life expectancy, functional status, and patient preferences. Recognizing the proportion and number of older diabetic adults in each of the 5 health status groups is the first step toward devising strategies to prevent worsening and perhaps to improve their health status.

Our state-specific synthetic estimates for the 5 health status groups are, to our knowledge, the first to correspond with the interventions in the PARG (11). Planners may also choose from other physical activity programs to balance the proportion and the total number of adults in each health status group against implementation needs and costs. For example, programs for the few homebound or frail adults will likely require more expensive trained staff to focus on improving and preserving physical function and range of motion; the homebound will likely require a program delivered in their homes, whereas the frail often need transportation to a facility where a program is held. Impaired adults may be able to use their own transportation to get to a community program facility. Adults who have comorbid conditions may require monitoring to avoid complications from physical activity programs. For example, older diabetic adults who use beta blockers to control hypertension may be less attentive to the symptoms of hypoglycemia than those not taking such medications, and their muted exercise heart rate response could lead to overexertion if they attempt to meet a traditional target heart rate applicable to healthy older adults (27). Diabetic adults who are otherwise healthy should be encouraged to be active to improve glycemic control, to avoid hypoglycemia, and to maintain physical function (5,24). Program planners should consider these and many other factors identified in the PARG to deliver safe and successful programs (11) for specific health status groups of inactive, older diabetic adults.

Our study had several limitations. We used the statistically conservative method of nonoverlapping confidence intervals to determine significance and assumed, within region, that states did not vary in their proportions of inactive, older, diabetic adults by specific health status group. This conservative approach has the advantage of partially controlling for multiple comparisons. We used self-report items of impairment and disability from NHIS rather than a detailed geriatric assessment protocol (28) that might better ascertain each health status. Because we used self-report for both health status groups and physical inactivity, the study's exposure (physical inactivity) and outcome (health status groups) are subject to misclassification. The outcome was not available in representative data sets. We also operationally defined the 5 health status groups primarily to correspond to terms found in the PARG (11). Some of the terms, such as "frail," are hard to define conceptually or operationally and are subject to various interpretations (29). Moreover, we did not replicate the multiple categories of disability recently outlined by Kalyani and colleagues (3), who also used NHIS data. They analyzed all people with diabetes, regardless of activity, which ruled out a comparison with our analyses of people with diabetes who were inactive. Another limitation is that we did not identify separate health status groups for the roughly 1 in 4 physically active, older diabetic adults. However, their need for physical activity programs is not as pressing as for the remaining inactive, older diabetic adults because the combined proportion of the homebound and the frail was higher for the inactive, 12.9% (homebound + frail = 3.3% + 9.6%, respectively), than for the active, 1.8% (homebound + frail = 0.5% + 1.3%, respectively). Nonetheless, active older adults with diabetes may benefit from a formal physical activity program. Lastly, we did not adjust our estimates for age or any other variable that might have produced some of the regional variation in our estimates because our goal was to present data for health status group by state. Age and other factors that differ among states might change the size of our estimates. However, because we stratified our analyses and presented data for only people who were physically inactive, we did reduce regional variation in health status groups.

In summary, among older, physically inactive, diabetic adults, the homebound, the frail, and the otherwise healthy were less common than those who were impaired or who had comorbid conditions. Tailored physical activity programs have much to offer older, physically inactive, diabetic adults. State program planners may use these estimates to select physical activity programs for each kind of health status group to enhance diabetes control and to prevent adverse health outcomes that lead to progressively worse impairment. Making such programs available may help to control the preventable burden of diabetes that is otherwise expected to grow unabated with the anticipated expansion of the older adult diabetic population. Because of this expansion, we anticipate that these state-level numbers may also change; therefore, future studies may investigate changes in these estimates as well as urban-rural and racial differences that may exist in health status groups among the states.

## Figures and Tables

**Table.** Characteristics of Respondents Aged ≥50 With Self-Reported Diabetes, 2000-2009 NHIS and 2000-2009 BRFSS

**Characteristic**	NHIS, % (95% CI) (n = 17,779)	BRFSS, % (95% CI) (n = 256,716)
**Age, y**		
50-64	49.6 (48.7-50.5)	48.9 (48.5-49.3)
65-74	28.6 (27.9-29.5)	29.0 (28.7-29.4)
75-84	17.8 (17.2-18.5)	19.1 (18.9-19.4)
≥85	3.9 (3.6-4.3)	3.0 (2.8-3.1)
**Male sex**	49.5 (48.6-50.5)	49.5 (49.1-49.9)
**Education**		
None	1.0 (0.9-1.2)	0.6 (0.5-0.7)
≤High school	60.2 (59.3-61.1)	48.8 (48.4-49.2)
>High school	38.8 (37.8-39.7)	50.6 (50.2-51.0)
**Race/ethnicity**		
Non-Hispanic white	69.4 (68.6-70.3)	67.8 (67.2-68.4)
Non-Hispanic black	14.8 (14.2-15.4)	13.8 (13.4-14.2)
Hispanic	11.2 (10.7-11.8)	11.1 (10.6-11.6)
Other	4.5 (4.1-4.9)	7.3 (6.9-7.7)

Abbreviations: NHIS, National Health Interview Survey; BRFSS, Behavioral Risk Factor Surveillance System; CI, confidence interval.

## Appendices

### Appendix A. State-specific proportions of physically inactive, diabetic adults aged 50 years or older, by health category, 2000-2009 NHIS and 2000-2009 BRFSS

This file is available for download as a Microsoft Word document. [DOC - 115k]

### Appendix B. State-specific numbers of physically inactive, diabetic adults aged 50 years or older, by health category, 2000-2009 NHIS and 2000-2009 BRFSS

This file is available for download as a Microsoft Word document. [DOC - 116k]
