# Supplementary material for: State-Specific Synthetic Estimates of Health Status Groups Among Inactive Older Adults With Self-Reported Diabetes, 2000-2009
Source: Prev Chronic Dis. 2012 Apr 19;9:E89. (PMC3406741)
Supplement: Supplementary file 1 [file 11_0221_01.doc]

| **Region/State** | **Size** | **Weighted n** | **Homebound** | | **Frail** | | **Impairment** | | **Comorbid** | | **Healthy** | |
| --- | --- | --- | --- | --- | --- | --- | --- | --- | --- | --- | --- | --- |
|  |  |  | **%** | **95% CI** | **%** | **95% CI** | **%** | **95% CI** | **%** | **95% CI** | **%** | **95% CI** |
| **Northeast** | | | | | | | | | | | | |
| Connecticut | 4083 | 128504 | 2.2 | (1.4-3.1) | 5.9 | (4.7-7.0) | 21.7 | (19.4-23.9) | 42.9 | (40.3-45.5) | 5.0 | (3.9-6.0) |
| Maine | 3379 | 58251 | 2.2 | (1.4-3.1) | 5.9 | (4.7-7.0) | 21.8 | (19.5-24.1) | 43.1 | (40.5-45.8) | 5.0 | (4.0-6.0) |
| Massachusetts | 9093 | 242578 | 2.2 | (1.4-3.1) | 5.8 | (4.7-7.0) | 21.6 | (19.4-23.8) | 42.8 | (40.3-45.3) | 5.0 | (3.9-6.0) |
| New Hampshire | 3580 | 48467 | 2.2 | (1.4-3.1) | 5.9 | (4.7-7.0) | 21.7 | (19.4-23.9) | 42.9 | (40.3-45.5) | 5.0 | (3.9-6.0) |
| New Jersey | 7462 | 380770 | 2.3 | (1.4-3.1) | 5.9 | (4.8-7.1) | 22.0 | (19.7-24.3) | 43.5 | (41.0-46.1) | 5.0 | (4.0-6.1) |
| New York | 4252 | 864707 | 2.2 | (1.4-3.1) | 5.9 | (4.8-7.0) | 21.8 | (19.6-24.1) | 43.2 | (40.6-45.8) | 5.0 | (4.0-6.0) |
| Pennsylvania | 7989 | 607319 | 2.3 | (1.4-3.1) | 6.0 | (4.8-7.1) | 22.1 | (19.8-24.4) | 43.7 | (41.2-46.3) | 5.1 | (4.0-6.1) |
| Rhode Island | 2995 | 43165 | 2.2 | (1.4-3.1) | 5.9 | (4.7-7.0) | 21.8 | (19.5-24.0) | 43.0 | (40.4-45.7) | 5.0 | (4.0-6.0) |
| Vermont | 3467 | 21631 | 2.2 | (1.4-3.1) | 5.9 | (4.7-7.0) | 21.8 | (19.5-24.0) | 43.1 | (40.5-45.7) | 5.0 | (4.0-6.0) |
| **Midwest** | | | | | | | | | | | | |
| Illinois | 3494 | 537970 | 2.3 | (1.6-3.1) | 6.9 | (5.7-8.2) | 25.1 | (23.1-27.1) | 38.1 | (35.9-40.4) | 4.1 | (3.2-5.0) |
| Indiana | 4755 | 282934 | 2.4 | (1.6-3.1) | 7.0 | (5.8-8.2) | 25.3 | (23.3-27.3) | 38.5 | (36.3-40.8) | 4.1 | (3.2-5.0) |
| Iowa | 3512 | 119547 | 2.4 | (1.7-3.2) | 7.2 | (5.9-8.5) | 26.1 | (24.0-28.2) | 39.7 | (37.4-42.0) | 4.2 | (3.3-5.2) |
| Kansas | 6268 | 106443 | 2.3 | (1.6-3.1) | 6.9 | (5.7-8.1) | 24.9 | (23.0-26.9) | 37.9 | (35.7-40.1) | 4.1 | (3.2-4.9) |
| Michigan | 5886 | 466688 | 2.3 | (1.6-3.1) | 6.8 | (5.6-8.1) | 24.8 | (22.8-26.7) | 37.7 | (35.5-39.8) | 4.0 | (3.1-4.9) |
| Minnesota | 2411 | 160671 | 2.4 | (1.6-3.1) | 7.0 | (5.8-8.3) | 25.4 | (23.4-27.5) | 38.7 | (36.3-41.0) | 4.1 | (3.2-5.0) |
| Missouri | 3962 | 249831 | 2.4 | (1.6-3.1) | 7.0 | (5.7-8.2) | 25.2 | (23.2-27.3) | 38.4 | (36.1-40.7) | 4.1 | (3.2-5.0) |
| Nebraska | 6692 | 67186 | 2.4 | (1.7-3.2) | 7.1 | (5.9-8.4) | 25.8 | (23.8-27.9) | 39.3 | (37.0-41.5) | 4.2 | (3.3-5.1) |
| North Dakota | 2543 | 24284 | 2.4 | (1.7-3.2) | 7.2 | (5.9-8.5) | 26.0 | (23.9-28.2) | 39.6 | (37.2-42.0) | 4.2 | (3.3-5.2) |
| Ohio | 6617 | 527395 | 2.3 | (1.6-3.0) | 6.8 | (5.6-8.0) | 24.6 | (22.7-26.6) | 37.5 | (35.3-39.7) | 4.0 | (3.1-4.9) |
| South Dakota | 4519 | 29074 | 2.4 | (1.6-3.2) | 7.1 | (5.8-8.3) | 25.6 | (23.5-27.6) | 38.9 | (36.6-41.2) | 4.2 | (3.2-5.1) |
| Wisconsin | 3376 | 207360 | 2.4 | (1.7-3.2) | 7.2 | (5.9-8.5) | 26.0 | (23.9-28.1) | 39.5 | (37.2-41.9) | 4.2 | (3.3-5.2) |
| **South** | | | | | | | | | | | | |
| Alabama | 4499 | 249325 | 2.9 | (2.3-3.4) | 8.5 | (7.6-9.3) | 22.5 | (21.2-23.9) | 37.1 | (35.6-38.7) | 5.1 | (4.4-5.8) |
| Arkansas | 3788 | 125653 | 2.8 | (2.2-3.4) | 8.3 | (7.5-9.2) | 22.1 | (20.8-23.4) | 36.4 | (34.8-38.0) | 5.0 | (4.3-5.7) |
| Delaware | 3053 | 35954 | 2.8 | (2.2-3.4) | 8.3 | (7.4-9.2) | 22.0 | (20.7-23.4) | 36.3 | (34.6-38.0) | 5.0 | (4.3-5.6) |
| DC | 2189 | 27187 | 2.9 | (2.3-3.5) | 8.6 | (7.7-9.5) | 23.0 | (21.5-24.4) | 37.8 | (36.0-39.6) | 5.2 | (4.4-5.9) |
| Florida | 10678 | 890505 | 3.0 | (2.3-3.6) | 8.8 | (7.9-9.7) | 23.3 | (21.9-24.7) | 38.4 | (36.8-40.0) | 5.2 | (4.5-6.0) |
| Georgia | 5363 | 393624 | 2.7 | (2.1-3.3) | 8.1 | (7.2-8.9) | 21.4 | (20.1-22.7) | 35.2 | (33.7-36.8) | 4.8 | (4.2-5.5) |
| Kentucky | 6908 | 200386 | 2.8 | (2.2-3.3) | 8.2 | (7.4-9.0) | 21.8 | (20.5-23.1) | 35.9 | (34.3-37.4) | 4.9 | (4.2-5.6) |
| Louisiana | 5379 | 211764 | 2.8 | (2.2-3.4) | 8.3 | (7.5-9.1) | 22.1 | (20.7-23.4) | 36.3 | (34.8-37.8) | 5.0 | (4.3-5.6) |
| Maryland | 5043 | 234901 | 2.8 | (2.2-3.4) | 8.4 | (7.5-9.3) | 22.3 | (21.0-23.7) | 36.7 | (35.2-38.3) | 5.0 | (4.3-5.7) |
| Mississippi | 6423 | 156159 | 2.7 | (2.2-3.3) | 8.2 | (7.3-9.0) | 21.7 | (20.4-23.0) | 35.7 | (34.2-37.2) | 4.9 | (4.2-5.5) |
| North Carolina | 10794 | 410906 | 2.9 | (2.3-3.5) | 8.6 | (7.7-9.4) | 22.8 | (21.4-24.1) | 37.5 | (36.0-39.0) | 5.1 | (4.4-5.8) |
| Oklahoma | 6666 | 169300 | 2.8 | (2.2-3.4) | 8.3 | (7.5-9.2) | 22.1 | (20.8-23.4) | 36.5 | (35.0-38.0) | 5.0 | (4.3-5.7) |
| South Carolina | 7165 | 216857 | 2.8 | (2.2-3.4) | 8.4 | (7.5-9.2) | 22.2 | (20.9-23.5) | 36.6 | (35.0-38.1) | 5.0 | (4.3-5.7) |
| Tennessee | 3755 | 306153 | 2.7 | (2.2-3.3) | 8.1 | (7.3-9.0) | 21.6 | (20.2-22.9) | 35.5 | (33.9-37.2) | 4.9 | (4.2-5.5) |
| Texas | 7211 | 946766 | 2.7 | (2.1-3.2) | 8.0 | (7.2-8.8) | 21.2 | (19.9-22.5) | 34.9 | (33.5-36.4) | 4.8 | (4.1-5.4) |
| Virginia | 3976 | 309617 | 2.9 | (2.3-3.5) | 8.6 | (7.7-9.5) | 22.9 | (21.5-24.3) | 37.7 | (36.0-39.4) | 5.1 | (4.4-5.9) |
| West Virginia | 3816 | 115317 | 2.9 | (2.3-3.5) | 8.6 | (7.8-9.5) | 23.0 | (21.6-24.3) | 37.8 | (36.3-39.4) | 5.2 | (4.5-5.9) |
| **West** | | | | | | | | | | | | |
| Alaska | 1029 | 16630 | 2.4 | (1.6-3.2) | 6.1 | (4.8-7.4) | 20.1 | (18.1-22.2) | 37.0 | (34.2-39.9) | 6.3 | (5.1-7.4) |
| Arizona | 3535 | 230089 | 2.5 | (1.7-3.3) | 6.4 | (5.1-7.7) | 21.1 | (19.1-23.1) | 38.7 | (36.2-41.3) | 6.6 | (5.4-7.7) |
| California | 4821 | 1464534 | 2.4 | (1.6-3.2) | 6.2 | (5.0-7.5) | 20.5 | (18.6-22.4) | 37.7 | (35.5-40.0) | 6.4 | (5.3-7.5) |
| Colorado | 3596 | 130363 | 2.5 | (1.7-3.3) | 6.4 | (5.1-7.7) | 21.1 | (19.2-23.1) | 38.9 | (36.5-41.3) | 6.6 | (5.4-7.7) |
| Hawaii | 3422 | 48258 | 2.6 | (1.7-3.4) | 6.6 | (5.3-7.9) | 21.8 | (19.8-23.8) | 40.0 | (37.6-42.5) | 6.8 | (5.6-8.0) |
| Idaho | 3494 | 50482 | 2.5 | (1.7-3.4) | 6.4 | (5.2-7.7) | 21.2 | (19.3-23.2) | 39.1 | (36.7-41.4) | 6.6 | (5.4-7.8) |
| Montana | 3474 | 32912 | 2.6 | (1.7-3.5) | 6.7 | (5.3-8.0) | 22.1 | (20.0-24.1) | 40.6 | (38.1-43.0) | 6.9 | (5.7-8.1) |
| Nevada | 2215 | 90560 | 2.4 | (1.6-3.3) | 6.3 | (5.0-7.5) | 20.6 | (18.7-22.6) | 37.9 | (35.4-40.5) | 6.4 | (5.3-7.6) |
| New Mexico | 4214 | 73136 | 2.5 | (1.7-3.3) | 6.4 | (5.1-7.6) | 21.0 | (19.1-23.0) | 38.7 | (36.3-41.0) | 6.5 | (5.4-7.7) |
| Oregon | 3444 | 138159 | 2.5 | (1.7-3.4) | 6.5 | (5.2-7.7) | 21.3 | (19.3-23.3) | 39.2 | (36.8-41.6) | 6.6 | (5.5-7.8) |
| Utah | 2997 | 66696 | 2.5 | (1.7-3.3) | 6.3 | (5.1-7.6) | 21.0 | (19.0-22.9) | 38.5 | (36.1-40.9) | 6.5 | (5.4-7.7) |
| Washington | 12588 | 227333 | 2.5 | (1.6-3.3) | 6.3 | (5.0-7.6) | 20.8 | (18.9-22.7) | 38.3 | (36.0-40.5) | 6.5 | (5.3-7.6) |
| Wyoming | 3116 | 17818 | 2.5 | (1.7-3.4) | 6.5 | (5.2-7.8) | 21.4 | (19.5-23.4) | 39.4 | (37.0-41.8) | 6.7 | (5.5-7.8) |
